# Aberrant protein S-nitrosylation contributes to hyperexcitability-induced synaptic damage in Alzheimer’s disease: Mechanistic insights and potential therapies

**DOI:** 10.3389/fncir.2023.1099467

**Published:** 2023-02-02

**Authors:** Swagata Ghatak, Tomohiro Nakamura, Stuart A. Lipton

**Affiliations:** ^1^School of Biological Sciences, National Institute of Science Education and Research, Bhubaneswar, India; ^2^Neurodegeneration New Medicines Center and Department of Molecular Medicine, The Scripps Research Institute, La Jolla, CA, United States; ^3^Department of Neurosciences, School of Medicine, University of California, San Diego, La Jolla, CA, United States

**Keywords:** hyperexcitability, Alzheimer’s disease, S-nitrosylation, NMDA receptors, glutamate excitotoxicity, NitroSynapsin

## Abstract

Alzheimer’s disease (AD) is arguably the most common cause of dementia in the elderly and is marked by progressive synaptic degeneration, which in turn leads to cognitive decline. Studies in patients and in various AD models have shown that one of the early signatures of AD is neuronal hyperactivity. This excessive electrical activity contributes to dysregulated neural network function and synaptic damage. Mechanistically, evidence suggests that hyperexcitability accelerates production of reactive oxygen species (ROS) and reactive nitrogen species (RNS) that contribute to neural network impairment and synapse loss. This review focuses on the pathways and molecular changes that cause hyperexcitability and how RNS-dependent posttranslational modifications, represented predominantly by protein S-nitrosylation, mediate, at least in part, the deleterious effects of hyperexcitability on single neurons and the neural network, resulting in synaptic loss in AD.

## 1. Introduction

Emerging evidence suggests that patients with Alzheimer’s disease (AD) manifest non-convulsive epileptic discharges, which are associated with a faster rate of cognitive decline (Vossel et al., 2013; Lam et al., 2017; Ghatak et al., 2019). This epileptiform activity in AD might arise as consequence of neuronal dysfunction during disease progression or it might be a part of an early AD phenotype, which leads to neurodegeneration. Both familial (F) and sporadic (S) AD patients show non-convulsive seizure activity with some evidence suggesting its presence in up to 42.4% of AD cases (Palop and Mucke, 2009, 2016). FAD patients with mutations in amyloid precursor protein (APP) or presenilin (PSEN or PS) genes 1/2, which increase amyloid-β (Aβ) peptide, show increased activation in the right anterior hippocampus by functional MRI early in the disease (Quiroz et al., 2010). Moreover, both humans with AD and AD transgenic murine models manifest spike-wave discharges (Verret et al., 2012; Vossel et al., 2013; Nygaard et al., 2015; Lam et al., 2017), with AD mouse models displaying impaired performance in behavioral tasks involving memory and spatial processing associated with covert epileptiform activity (Scharfman, 2012a,b; Verret et al., 2012; Chin and Scharfman, 2013). In this review, we outline the basis for this abnormal hyperelectrical activity as well as emerging treatment modalities to reverse it and thus abate cognitive decline.

## 2. Network abnormalities in AD

Non-convulsive seizure activity in AD is associated with neuronal hyperexcitability. Excitability changes occur in several brain structures, with early hyperactivity initiated in the dentate gyrus (Palop et al., 2007), and then spreading to the hippocampus (Dickerson et al., 2005; Palop et al., 2007). Subsequently, functionally and structurally connected regions of the brain become involved as AD pathology spreads (Leal et al., 2017; Busche and Hyman, 2020). Since higher brain regions involved in learning and memory depend on the interaction of neurons from local neuronal microcircuits to larger/long-range networks, neuronal hyperactivity that disrupts micro- and macro-scale network function can lead to more rapid disease progression and therefore cognitive disability in AD patients (Wang et al., 2010; Vossel et al., 2016). Accordingly, AD is now conceptualized as brain network disorder.

Contributing types of network disruption include network hypersynchrony, activation and deactivation deficits, and abnormal oscillatory activity (Palop and Mucke, 2016; Martinez-Losa et al., 2018). As these functional changes in the neural network overlap with the brain regions that eventually manifest pathological hallmarks of AD, they may act as an early indicator of disease and could potentially be a causal factor contributing to the manifestation of clinical disease (Palop and Mucke, 2016).

Cognitive function is influenced by different brain states, which in turn reflect distinct modes of neural activity (Boly et al., 2008). Different modes of brain activity are in part dependent on the degree of synchrony in firing among neuronal populations, a phenomenon called network synchrony (Jasper, 1936). Non-active states, such as slow-wave sleep or quiet wakefulness, can be distinguished from active states by the difference in network synchrony. During non-active states, neuronal activity is synchronized at different sites of the cortex, with slow fluctuations of high amplitude. Contrary to this, during active behaviors, such as paying attention or learning, neuronal activity at different sites becomes desynchronized, with fluctuations of higher frequencies but smaller amplitudes (Destexhe et al., 1999; Palop and Mucke, 2016; Poulet and Crochet, 2018). Interestingly, the different functional states of the brain, and the corresponding synchrony in neuronal activity, are correlated in various brain regions such as the hippocampus and neocortex (Buzsaki, 2002; Kay and Frank, 2019). This suggests that network synchrony is fundamental for normal brain function and any disruption in synchrony could be an important pathogenic mechanism underlying AD-related cognitive dysfunction (Uhlhaas and Singer, 2006). Additionally, we know that when a healthy individual performs a cognitively demanding task, the brain region required for that particular task becomes more active while other brain regions undergo large scale deactivation, termed default mode network (DMN) deactivation (Raichle et al., 2001; Boyatzis et al., 2014). Accordingly, poor memory formation has been shown to be related to task-induced hippocampal activation without adequate DMN deactivation, highlighting the dependence of proper execution of complex functions on well-coordinated neuronal network activity (Filippini et al., 2009; Anticevic et al., 2012).

Although AD is increasingly thought of as a heterogeneous, multifactorial disorder, network abnormalities, especially in the early stages of the disease, appear to be consistent across several models of AD, including transgenic murine models, human induced pluripotent stem cell (hiPSC)-derived neuronal models, and also in AD patients (Vossel et al., 2013, 2016, 2017; Siskova et al., 2014; Lam et al., 2017; Ghatak et al., 2019, 2021a). Patients with both mild cognitive impairment (MCI), who are at risk for developing AD at later timepoints, and presymptomatic carriers of FAD mutations, who are destined to develop AD, manifest hippocampal hyperactivation and reduced deactivation of DMN components during memory-encoding tasks (Bookheimer et al., 2000; Dickerson et al., 2005; Celone et al., 2006; Quiroz et al., 2010). Interestingly, it has been observed that this network dysfunction also occurs in cognitively normal people with cerebral amyloid deposits (Sperling et al., 2009). Although early hippocampal and cortical hyperactivation has been interpreted as a compensation mechanism for emerging cognitive decline in AD patients (Kunz et al., 2015), accumulating evidence suggests that this hyperactivation contributes to cognitive decline and is an important component of AD pathogenesis (Putcha et al., 2011). Moreover, arguably the best neuropathological correlate to cognitive decline in AD is synaptic loss (DeKosky and Scheff, 1990; Terry et al., 1991), and, as discussed below, we have shown that molecular pathways triggered at least in part by aberrant hyperactivity, can contribute to this synaptic loss (Ghatak et al., 2021a,b; Nakamura et al., 2021a,b).

Analysis of the power spectrum of spontaneous calcium transients in AD hiPSC derived neuronal/glial cultures compared to isogenic wild-type revealed increased bursts of low-frequency (< 1 Hz) events (Ghatak et al., 2021a). These low frequency events contribute to very slow oscillations (in the 0.2–1 Hz range) that constitute the default cortical activity pattern as observed *in vivo* (Krishnan et al., 2018). The peaks of neuronal calcium transients potentially coincide with the “upstate” of these slow oscillations, and the silent troughs reflect the “downstate”(Sanchez-Vives et al., 2017). Greater spontaneous calcium transients, as observed in AD hiPSC derived neurons, potentially indicate a prolonged upstate and shortening of the downstate, which could potentially cause network dysfunction (Ghatak et al., 2021a). Mechanisms underlying such hyperactivation and increased slow oscillations in cortical and hippocampal networks in AD indicate a change in the excitatory to inhibitory (E/I) ratio at the synaptic and neuronal network level, resulting in E/I imbalance.

Additionally, aberrant gamma band (∼40 Hz) oscillations, with reduced power and synchronization, have been observed in transgenic AD models and in human AD brain on spectral analysis of EEG. Intriguingly, recent evidence suggests that entrainment of gamma with sensory stimuli may improve AD pathology and cognitive performance (Iaccarino et al., 2016; Adaikkan et al., 2019). Gamma band oscillations occur in multiple brain regions including the hippocampus and are thought to be important in selective attention and memory operations, including maintenance of working memory (van Vugt et al., 2010; Mably and Colgin, 2018). With gamma band oscillations dependent particularly on inhibitory synaptic transmission (Buzsaki and Wang, 2012), the synaptic damage and E/I imbalance of AD can be important contributors to the development of these defective oscillations.

## 3. Development of E/I imbalance in AD

**FIGURE 1:**
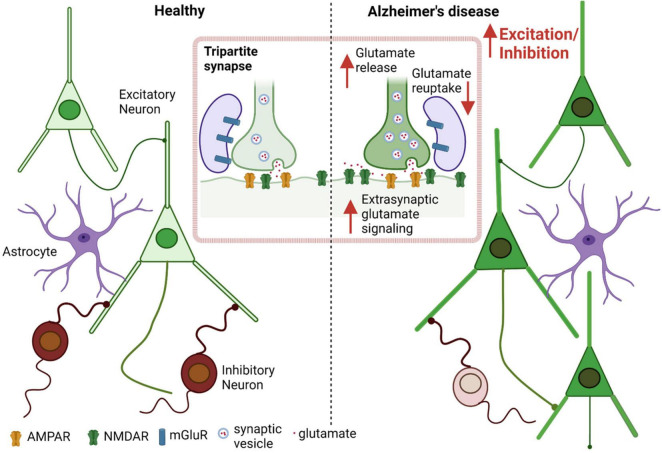
Aberrant glutamate signaling contributes to E/I imbalance in AD. Increased excitation and decreased inhibition leads to an increased E/I ratio in AD as well as in other neurological diseases such as autism spectrum disorder (ASD) (Ghatak et al., 2021b). Excessive presynaptic glutamate release, disrupted glutamate re-uptake or frank release by astrocytes, and resulting increased (predominantly extrasynaptic) NMDA receptor-mediated signaling leads to increased excitation at the single neuron level, contributing to hyperexcitability in the neuronal network.

Several AD transgenic murine models have shown Aβ-induced changes in E/I balance, with resulting cortical and hippocampal neuronal hyperexcitability. Additionally, these models display disruption of slow-wave oscillations (and fast-wave gamma oscillations, as mentioned previously) with increased network hypersynchrony, even before the appearance of amyloid plaques (Busche et al., 2008; Rudinskiy et al., 2012; Maier et al., 2014; Busche and Konnerth, 2016). Balance between total excitation and inhibition within a network is integral to normal cognition and memory, which is maintained by an appropriate excitatory to inhibitory synaptic input ratio at the individual neuron level, and by regulating the interaction between various excitatory and inhibitory neurons at the circuit level (Barral and Reyes, 2016; Ghatak et al., 2021b; Lauterborn et al., 2021). In addition to intrinsic regulation of electrical activity caused by properties like resting membrane potential, firing threshold, input resistance etc., factors extrinsic to the cell, including GABA, glutamate, and the presence of misfolded proteins such as Aβ oligomers, can modulate the E/I ratio (Abramov et al., 2009; Styr and Slutsky, 2018). Evidence suggests that Aβ oligomers are associated with neuronal circuit hyperactivity in early stages of AD, mediated by both increased excitation and decreased GABAergic inhibition (Palop et al., 2007; Abramov et al., 2009; Palop and Mucke, 2010; Talantova et al., 2013; Ghatak et al., 2019). Prior studies indicate that this increased excitation is due at least in part to dysfunction in the glutamatergic system in the cortex and hippocampus (Danysz and Parsons, 2012; Ghatak et al., 2019; Yuan et al., 2022). Elevated glutamate levels in the cerebrospinal fluid (CSF), increased glutamate receptor expression and activity, and decreased glutamate clearance as well as increased release, have all been found to contribute to the neuronal hyperactivation observed in AD (Figure 1; Li et al., 2009; Pirttimaki et al., 2013; Talantova et al., 2013; Madeira et al., 2018; Ghatak et al., 2019; Zott et al., 2019; Ortiz-Sanz et al., 2022).

### 3.1. Abnormalities in glutamate release and clearance in AD

Glutamate clearance (predominantly by astrocytes) and recycling (via the glutamate/glutamine shuttle, also in astrocytes) are important factors that determine the availability of glutamate as a neurotransmitter for proper signaling and prevention of neuronal hyperexcitation. In AD, this system is severely affected with a decrease in glutamate transporter capacity (Masliah et al., 1996; Li et al., 1997). To account for this finding, prior studies have shown that Aβ peptide oligomers decrease glutamate re-uptake in neuronal cell cultures as well as *in vivo* in the CA1 hippocampal region of mice (Li et al., 2009; Zott et al., 2019). Additionally, Aβ_42_ oligomers have been shown to cause release of glutamate from astrocytes, contributing to neuronal hyperexcitability (Talantova et al., 2013).

After synaptic release of glutamate, the level of the transmitter is quickly regulated back to baseline by re-uptake into astrocytes (Conway and Hutson, 2016; Yudkoff, 2017). At rest, the concentration of glutamate is ∼0.6 μM (Bouvier et al., 1992), but transiently increases to approximately 1 mM in the synaptic cleft following action potential-mediated neuronal depolarization and release (Clements et al., 1992; Dzubay and Jahr, 1999). Glutamate is removed by specific transporters (GLAST/EAAT1 (glutamate-aspartate transporter/excitatory amino acid transporter 1) and GLT-1 (glutamate transporter-1)/EAAT2) (Masliah et al., 1996). Due to disruption of glutamate clearance and excessive release in AD, pathological accumulation of glutamate occurs, which overstimulates glutamate receptors (predominantly extrasynaptic *N-*methyl-D-aspartate (NMDA)-type glutamate receptors) on neurons and causes excessive calcium entry and downstream injury to synapses (Choi et al., 1987; Sattler and Tymianski, 2000; Mattson and Chan, 2003; Pirttimaki et al., 2013; Talantova et al., 2013; Zott et al., 2019).

Concerning the specific transporter involved, EAAT2 (GLT-1) in astrocytes takes up the majority of extracellular glutamate (Lehre and Danbolt, 1998; Scott et al., 2011). EAAT2 protein levels are decreased in AD patients, which leads to a decrease in glutamate clearance by astrocytes (Masliah et al., 1996; Ikegaya et al., 2002; Scott et al., 2002, 2011; Jacob et al., 2007), but see (Li et al., 1997). Moreover, not only is glutamate re-uptake affected, but glutamate release has been shown to be increased with real-time imaging techniques using a FRET-based sensor. This effect on glutamate release was demonstrated to be mediated by Aβ oligomers interacting with α7 nicotinic acetylcholine receptors on astrocytes (Talantova et al., 2013), an effect confirmed by others (Pirttimaki et al., 2013). In addition to increasing astrocytic glutamate release, Aβ has been reported to increase release probability at neuronal presynaptic terminals (Abramov et al., 2009). In hiPSC-derived AD neurons, increased levels of vesicular glutamate transporter 1 (VGLUT1) have been observed, which may underlie the increase in glutamate release from presynaptic terminals (Ghatak et al., 2019).

Persistently elevated glutamate interacts with postsynaptic receptors leading to aberrant neuronal depolarization, increased calcium levels, and activation of downstream pathways leading to excitotoxic dysfunction; these processes can lead to synaptic damage, as described below, and eventually to neuronal cell death (Hardingham et al., 2002; Talantova et al., 2013; Ghatak et al., 2019). The major postsynaptic glutamate receptors are comprised of α-amino-3-hydroxy-5-methyl-4-isoxazolepropionic acid (AMPA) receptors, NMDA receptors, and metabotropic glutamate receptors (mGluRs), which may be affected to different extents in AD, and contribute to the pathology. AMPA receptors are also critical to normal NMDA receptor function in that the initial ionic influx in response to glutamate-activated AMPA receptor-operated channels is known to depolarize neurons, thereby relieving physiological Mg^2+^ block of NMDA receptor-operated channels (Nowak et al., 1984).

### 3.2. AMPA receptor dysfunction in AD

AMPA receptor dysfunction correlates with the presence of soluble Aβ oligomers. For example, Aβ is known to bind to the C-terminal region of the GluA2 subunit of calcium-impermeable AMPA receptors leading to internalization of these receptors (Lacor et al., 2004; Zhao et al., 2004, 2010). This induces synaptic modifications via increases in ubiquitination, internalization, and degradation of AMPA receptors (Passafaro et al., 2003; Zhang et al., 2018); the effect of redox posttranslational modification of AMPA receptors and other glutamate-related proteins is discussed below after we introduce the effects of nitric oxide (NO)-related species on this redox process. Additionally, Ca^2+^ permeable AMPA GluA2 subunit-containing receptors have been shown to contribute to excitotoxicity and could play a role in AD related E/I imbalance (Whitehead et al., 2017).

Various modulators, such as Arc (activity-regulated cytoskeletal gene), control AMPA receptor surface expression and subunit composition, and maintain homeostatic control of the optimal level required for normal neuronal plasticity and physiology (Gao et al., 2010; Guo and Ma, 2021). If Arc-mediated endocytosis remains unchecked, excessive modification of synaptic strength might generate instability or altered synchrony in neuronal networks, leading to disease states characterized by network imbalances, as observed in AD (Shepherd et al., 2006; Kerrigan and Randall, 2013). Along these lines, activity dependent expression of Arc is known to be disrupted in the cortex of APP/PS1 AD mice (Rudinskiy et al., 2012).

mGluRs are another potentially important contributor to excitotoxic damage in AD (Srivastava et al., 2020). Of the 8 types of mGluRs, mGluR1 and mGluR5 levels have been shown to be decreased in cerebral cortex and hippocampus of AD patients and AD transgenic murine models (Albasanz et al., 2005; Fang et al., 2017; Bie et al., 2019; Srivastava et al., 2020). mGluR5 is a transmembrane G-protein-coupled receptor linked to phospholipase C and inositol 1,4,5-trisphosphate and increases intracellular calcium levels following its activation (Jong et al., 2009). Enhanced binding of Aβ oligomers to mGluR5 in association with cellular prion proteins has been reported to result in impaired lateral diffusion and enhanced clustering of the receptor, leading to excessive release of intracellular Ca^2+^ and excitotoxicity (Um et al., 2013; Abd-Elrahman et al., 2020). Accordingly, genetic deletion of mGluR5 in the APP/PS1 murine model of AD prevented Aβ-related neuropathology and memory loss (Abd-Elrahman et al., 2020). Moreover, several studies have suggested a potential therapeutic role of mGluR5 in AD (Spurrier et al., 2022).

### 3.3. NMDA receptor dysfunction in AD

In addition to AMPA receptors and mGluRs, NMDA receptors play arguably the most important role in excitotoxic damage and E/I imbalance. While glutamatergic neurotransmission via synaptic NMDA receptors is critical for induction of long-term potentiation (LTP) and survival of neurons in several brain areas, excessive NMDA receptor activity (especially extrasynaptic NMDA receptors) contributes to aberrant gene transcription and excitotoxic pathways leading to synaptic damage, neurodegeneration and cognitive decline in AD and in other neurologic diseases (Hardingham et al., 2002; Lipton, 2006; Alberdi et al., 2010; Danysz and Parsons, 2012; Talantova et al., 2013; Wang and Reddy, 2017; Ghatak et al., 2021b; Ortiz-Sanz et al., 2022). In contrast, physiological synaptic NMDA receptor activity leads to gene expression that opposes these destructive processes (Hardingham et al., 2002; Hardingham and Bading, 2010). Synaptic plasticity dictates cognitive function to a large extent. For example, LTP is associated with synaptic strengthening, whereas repetitive long-term depression (LTD) can be associated with synapse loss (Malinow and Malenka, 2002; Citri and Malenka, 2008; Shinoda et al., 2010; Bliss et al., 2014). During LTP induction, glutamate release from the presynaptic terminals activates AMPA receptors leading to depolarization of the postsynaptic terminal and removal of Mg^2+^ blockade from NMDA receptor-operated channels (Bliss and Collingridge, 1993; Malinow and Malenka, 2002; Citri and Malenka, 2008). This NMDA receptor stimulation mediates influx of Ca^2+^ and triggers a Ca^2+^/calmodulin-dependent protein kinase II (CaMKII)-mediated signaling cascade, which enhances synaptic strength (Bliss and Collingridge, 1993; Malinow and Malenka, 2002). On the other hand, tonic activation of NMDA receptors triggers phosphatase-mediated LTD and induces dendritic spine shrinkage (Luscher and Malenka, 2012). Specifically, tonic activation of extrasynaptic NMDA receptors has been shown to be involved in sustained increases in Ca^2+^, which contribute to LTD (Papouin and Oliet, 2014). In models of AD, pathological Aβ oligomers are known to impair LTP (Walsh et al., 2002; Walsh and Selkoe, 2004; Cleary et al., 2005) and enhance LTD (Kim et al., 2001; Hsieh et al., 2006; Li et al., 2009). Aβ-induced LTD may involve receptor internalization and subsequent collapse of dendritic spines (Hsieh et al., 2006; Shankar et al., 2007; Palop and Mucke, 2010). Additionally, Aβ oligomers are have been shown to aberrantly activate extrasynaptic NMDA receptors, which in turn can contribute to LTD as well as to synaptic loss (Talantova et al., 2013; Ghatak et al., 2021a,b).

In addition to impairment of LTP by Aβ oligomers via dysfunctional synaptic NMDA receptors, there are other pathological mechanisms involving NMDA receptors that lead to synaptotoxicity and neurodegeneration (Tu et al., 2014). For example, soluble Aβ oligomers can impair glutamate re-uptake mechanisms or potentiate release to increase extracellular glutamate levels and thereby activate extrasynaptic NMDA receptors that contribute to neuronal hyperexcitability (Talantova et al., 2013; Lei et al., 2016). NMDA receptors containing GluN2B (the predominant subunit in extrasynaptic NMDA receptors) have been shown to directly contribute to neuronal excitability and seizures (Ying et al., 2004; Moddel et al., 2005). In part accounting for these deleterious effects, while physiological stimulation of synaptic NMDA receptors leads to neuroprotection via activation of cell survival genes like CREB, suppression of cell death pathways, and enhancement of intrinsic antioxidant defenses, excessive extrasynaptic NMDA receptor activation triggers pro-death signaling pathways, including downregulation of CREB-mediated gene transcription, upregulation of FOXO-mediated gene transcription, and ERK1/2 inactivation (Hardingham et al., 2002; Hardingham and Bading, 2010; Parsons and Raymond, 2014).

Recently, we reported increased glutamate-evoked responses in AD patient hiPSC-derived cerebrocortical neurons compared to isogenic wild-type controls. These large glutamate responses were inhibited by the FDA approved drug, memantine, which our laboratory previously developed (Lipton, 2006) and to a much greater extent by an equimolar concentration of the improved NMDA receptor antagonist NitroSynapsin (Lipton, 2006, 2007; Ghatak et al., 2021a,b) (see section 4.1 below for the mechanism of action of NitroSynapsin). Interestingly, both memantine and NitroSynapsin spared normal transmitter-induced activity in wild-type neurons (Ghatak et al., 2021a). Accounting for this effect, aminoadamantane drugs in this class are known to preferentially block extrasynaptic (GluN2B-predominant) NMDA receptor-operated channels, which are pathologically open for longer periods of time, over physiological/phasic opening of synaptic channels (Chen et al., 1992; Chen and Lipton, 1997; Lipton, 2006; Ferreira et al., 2012; Takahashi et al., 2015). Accumulating evidence also suggests that glutamate (particularly AMPA) receptors may be directly dysregulated by Aβ oligomers, resulting in disruption of glutamatergic synaptic transmission in parallel to the development of early cognitive deficits (Hsieh et al., 2006).

In contrast to functional alterations in NMDA receptors, studies on expression of NMDA receptor subunits in the AD brain have been largely inconclusive. In some studies, levels of GluN1 (the major NMDA receptor subunit) were decreased in AD brain, while others found it to be unchanged (Hynd et al., 2001; Sze et al., 2001; Bi and Sze, 2002; Jacob et al., 2007). A few studies have reported that levels of both GluN2A and GluN2B NMDA receptor subunit mRNA and protein were decreased in AD brain (Bi and Sze, 2002; Hynd et al., 2004), while levels of GluN2C and GluN2D mRNA did not differ from that of controls (Hynd et al., 2004). Nonetheless, other evidence shows increased GluN2A and GluN2B expression in certain regions of the hippocampus and cortex in AD brain (Yeung et al., 2021). Recent studies have found enhanced expression of GluN2B in postsynaptic density fractions of prefrontal cortex from early-stage AD patients (Ortiz-Sanz et al., 2022). These discrepancies suggest that the expression of NMDA receptor subunits in AD varies according to brain regions and may be differentially affected in depending on stage of disease.

A major factor that contributes to composition and function of various glutamate receptors is posttranslational modification. For example, our group has mounted evidence that redox-mediated posttranslational modifications affect NMDA receptor activity. Additionally, redox-active molecules generated in response to excessive NMDA receptor stimulation in AD brain, particularly aberrant extrasynaptic NMDA receptors, regulate a large number of pathways involved in the pathogenesis of AD, contributing, for example, to synaptic damage (Cho et al., 2009; Nakamura et al., 2010; Qu et al., 2011; Talantova et al., 2013; Okamoto et al., 2014; Tu et al., 2014). Accounting for most of these effects downstream of excessive NMDA receptor activity, an exemplary redox modification of proteins involving NO-related signaling is considered below.

## 4. NO signaling and protein S-nitrosylation in AD

Emerging evidence suggests that advanced age, pathologically-aggregated proteins, neuroinflammation, the environmental exposome, as well as intrinsic aberrant hyperexcitability all contribute to excessive accumulation of reactive nitrogen species (RNS) and reactive oxygen species (ROS) (Nakamura et al., 2013). While the exposome is comprised of various reactive and inflammatory substances, NOx (representing RNS) and small particulate matter (which stimulates the production of endogenous RNS) have been shown in epidemiological studies to be an especially prominent factor (Alemany et al., 2021; Mork et al., 2022). These insults are thought to occur in several neurodegenerative diseases, including AD, Parkinson’s disease (PD)/Lewy body dementia (LBD), frontotemporal dementia (FTD), and HIV-associated neurocognitive disorder (HAND) (Barnham et al., 2004; Nakamura et al., 2013, 2021a,b; Doulias et al., 2021). Due to its reactive chemical nature, high concentrations of ROS/RNS aberrantly interact with a multitude of cellular molecules, triggering signaling cascades that lead to abnormal neuronal and synapse function. Thus, excessive amounts of ROS/RNS can interfere in many aspects of normal brain function. In contrast, under physiological conditions, production of ROS/RNS is tightly controlled, and cellular redox states are well-balanced by the intracellular antioxidant system. Low levels of ROS/RNS affect regulatory signaling pathways promoting synaptic plasticity as well as neuronal differentiation and survival (Lipton et al., 1993; Tenneti et al., 1997; Mannick et al., 1999). Hence, depending on the amount, subcellular location, and timing of the production, ROS/RNS plays important roles in both normal and aberrant cell signaling in health and disease (Stamler et al., 2001; Barnham et al., 2004; Hess et al., 2005; Nakamura et al., 2013, 2021a,b; Lundberg and Weitzberg, 2022). Here, we focus on aberrant redox-mediated, NO-dependent posttranslational modifications of critical cysteine residues, known as protein S-nitrosylation, which regulate cellular events associated with hyperexcitability and synaptic damage in AD.

NO synthases (NOS) catalyze production of NO via conversion of L-arginine to L-citrulline (Bredt and Snyder, 1994). Three NOS isoforms are present in the mammalian system and named based on their activity or location by cell type. Among them, two of the isoforms (i.e., neuronal NOS [nNOS or NOS1] and endothelial NOS [eNOS or NOS3]) synthesize NO in a calcium-dependent manner, whereas the third isoform, inducible NOS (iNOS or NOS2) is transcriptionally controlled and predominantly located in microglia and astrocytes, particularly during neuroinflammatory changes as observed in AD. In neurons, the NMDA receptors form a protein complex with PSD-95 and nNOS (Sattler et al., 1999). Stimulation of NMDA receptors triggers Ca^2+^ influx into the neuron through its associated ion channel, resulting in nNOS activation. While early studies found that NO exerts its biological function through stimulation of soluble guanylyl cyclase (sGC), promoting production of cyclic guanosine monophosphate (cGMP) (Nisoli et al., 2003), more evidence from our group, that of Jonathan Stamler, and subsequently others have demonstrated that an even more common mediator of NO signaling involves protein S-nitrosylation under both physiological and pathological conditions (Hess et al., 2005).

Multiple chemical pathways may potentially contribute to S-nitrosothiol formation and thus protein S-nitrosylation in a cellular context. Mechanistically, these include but not limited to (*i*) direct reaction of NO*^•^* with thiyl radical (R-S*^•^*); (*ii*) oxidation of NO*^•^* to an NO group ‘intermediate’ that possesses NO^+^-like character (e.g., as found in N_2_O_3_, or via complex with a transition metal as an acceptor of the electron in the outer pi molecular orbital of NO*^•^* to produce NO^+^-like character) followed by reaction with thiolate anion (R-S^–^), where “R” represents a peptide/protein containing a reactive cysteine residue; and (*iii*) transnitros(yl)ation, representing transfer of an NO group from one protein thiolate to another in a kinetically and thermodynamically favorable environment (Smith and Marletta, 2012; Nakamura and Lipton, 2013; Lancaster, 2017; Nakamura et al., 2021a,b). In the case of protein-protein transnitrosylation, R-S^–^ on the recipient protein initiates a reversible nucleophilic attack on the nitroso nitrogen of the donor S-nitrosylated protein (R_1_-SNO) to produce transfer of the NO group to a second protein (R_2_-SNO) (Hess et al., 2005; Smith and Marletta, 2012; Nakamura et al., 2021a,b). The physiological relevance of protein-S-nitrosylation was first reported on the NMDA receptor in the brain (Lei et al., 1992; Lipton et al., 1993). Subsequent studies, including recent S-nitroso-proteomics analyses, demonstrated the ubiquitous nature of protein *S*-nitrosylation, occurring on a multitude of targets, particularly under pathological conditions (Stamler et al., 2001; Hess et al., 2005; Nakamura et al., 2013, 2021a,b; Lundberg and Weitzberg, 2022). It should be noted that another reaction of RNS, involving NO*^•^* reacting with superoxide anion (O_2_*^•^*^–^), results in a formation of peroxynitrite (ONOO^–^), which contributes to nitration of tyrosine residues on proteins including α-synuclein and Aβ, enhancing aberrant protein aggregation (Ischiropoulos et al., 1992).

## 5. S-Nitrosylation regulates NMDA receptor activity and hyperexcitability

Cysteine thiols are often located in a critical region of proteins important for their function. For example, reactive cysteines often reside in catalytic or allosteric sites that can control enzymatic activity, ligand- or effector-binding sites on ion channels, DNA binding of transcription factors, and interaction domains between proteins or with their molecular chaperones (Hess et al., 2005; Nakamura et al., 2013, 2021a,b; Okamoto et al., 2014). Hence, S-nitrosylation of these cysteines can significantly affect the intrinsic activity of the given protein.

**FIGURE 2:**
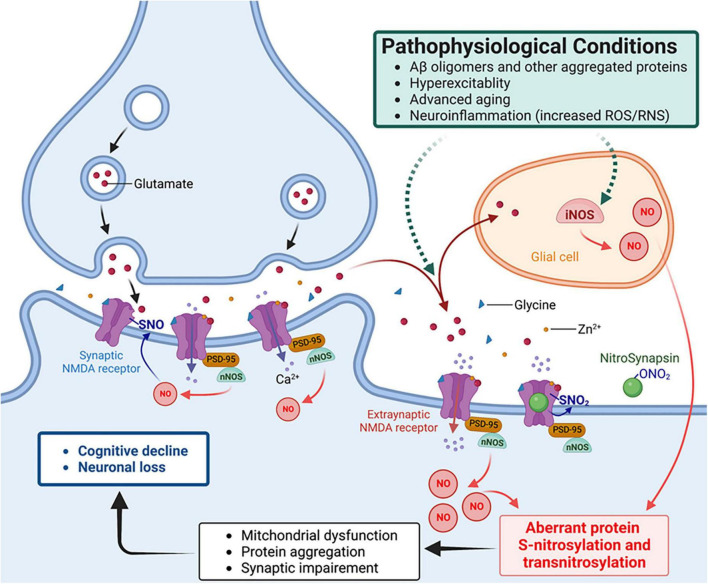
NitroSynapsin ameliorates aberrant S-nitrosylation that contributes to the pathophysiology of hyperexcitability and synaptic dysfunction in Alzheimer’s disease (AD). Oligomerized amyloid-β peptide (Aβ), neuronal hyperexcitability, aggregated proteins, and neuroinflammation can each trigger excessive NO production via inducible or neuronal NO synthase (glial iNOS or neuronal nNOS – the latter is physically tethered to the NMDA receptor), resulting in aberrant protein S-nitrosylation. The ensuing abnormal signaling mediated by protein S-nitrosylation and transnitrosylation results in mitochondrial fragmentation, bioenergetic compromise, and consequent synaptic impairment (Nakamura et al., 2021a,b). NitroSynapsin is a unique drug candidate that manifests dual actions to inhibit excessive (mainly extrasynaptic) NMDA receptor activity, thereby limiting subsequent NO production and its consequent downstream damage to synapses. Mechanistically, NitroSynapsin blocks the receptor’s ion channel when it is excessively open through its aminoadamantane moeity, and then delivers a nitro (–NO_2_) group specifically to the NMDA receptor at sites of S-nitrosylation to further decrease excessive receptor activity. SNO, S-nitrosylation or S-nitrosothiol; PSD-95, postsynaptic density protein 95.

One of best-characterized mechanisms of action for an SNO-protein entails negative regulation of NMDA receptor activity (Lei et al., 1992; Lipton et al., 1993, 2002; Choi et al., 2000; Takahashi et al., 2007). As alluded to above, physiological activation of synaptic NMDA receptors drives the production of NO at levels sufficient to maintain normal synaptic plasticity and promotes neuronal differentiation and survival. In contrast, activation of extrasynaptic NMDA receptors, for example, by oligomeric Aβ, aggregated α-synuclein or other stimulatory pathways, can generate excessive amounts of RNS that contribute to aberrant gene transcription, neurodegenerative phenotypes, such as mitochondria dysfunction, and further protein aggregation (Ryan et al., 2013; Talantova et al., 2013; Molokanova et al., 2014; Figure 2). Aberrant protein S-nitrosylation or transnitrosylation of a large number of proteins represents a pathological signaling event mediating many neurodegenerative effects of NO (Nakamura et al., 2013, 2021a,b). In contrast, S-nitrosylation of NMDA receptors is a physiological negative-feedback mechanism, whereby S-nitrosylation inhibits excessive receptor activity, suppressing hyperexcitability and synaptic damage (Lipton et al., 1993, 2002; Takahashi et al., 2007; Ghatak et al., 2021a).

Neuroprotective effects of S-nitrosylation-mediated inhibition of NMDA receptors are mediated via chemical reaction on thiol groups in at least five cysteine residues: Cys744 and Cys798 on the GluN1 subunit; Cys87, Cys320, and Cys399 on the GluN2A subunit (and presumably on the homologous residues at Cys86 and Cys321 on the GluN2B subunit, although this remains to be studied) (Choi et al., 2000). Under highly oxidizing conditions (such as exposure to room air with 150 torr of oxygen), Cys744 and Cys798 on the GluN1 subunit and Cys87 and Cys 320 on the GluN2A subunit can form disulfide bonds. However, in a less oxidizing environment, as seen under normal brain oxygen tension (∼11-50 torr), free thiols are favored. Moreover, pathological hypoxia (< 8-10 torr) further increases the proportion of free thiol groups at Cys744 and Cys798 on the GluN1 subunit, facilitating S-nitrosothiol formation at these sites (Takahashi et al., 2007). Along these lines, using x-ray crystallographic studies, we verified that Cys744 and Cys798 on GluN1 are susceptible to S-nitrosylation once the disulfide bond between them is reduced (Takahashi et al., 2007).

Mechanistically, S-nitrosothiol formation at Cys744 and/or Cys798 on the GluN1 subunit, which occurs preferentially under hypoxic conditions, sensitizes GluN2A(Cys399) to also undergo S-nitrosylation, possibly via a conformational change (for a schematic illustration of the effect of hypoxia on S-nitrosylation of Cys399, see Figure 8 in Takahashi et al. (2007)). Importantly, substitution of these cysteine residues to alanines on GluN1 and GluN2A prevented the inhibitory action of NO on the NMDA receptor, demonstrating that reaction of these cysteine residues accounts for the effect (Choi et al., 2000; Takahashi et al., 2007). The S-nitrosylation of Cys399 on GluN2A facilitates enhanced binding of glutamate and Zn^2+^ to their cognate binding domains on the GluN2A subunit. As a consequence, receptor desensitization results, thus abating NMDA receptor-mediated hyperexcitability and subsequent synaptic damage (Lipton et al., 2002).

### 5.1. NitroSynapsin inhibits NMDA receptor activity and thus hyperexcitability

Based on S-nitrosylation of NMDA receptors, one approach to decrease various pathogenic features of AD, including protein aggregation, mitochondrial impairment, neuronal hyperexcitability, and synaptic damage, would be selective S-nitrosylation of excessively activated NMDA receptors. Specifically, we hypothesized that selective suppression of hyperactivated NMDA receptors (particularly extrasynaptic receptors) by targeted S-nitrosylation would ameliorate Ca^2+^ influx through the channel thereby inhibiting aberrant downstream NO signaling.

Previously, our group spearheaded the clinical development of the FDA-approved drug memantine (in the form of Namenda^®^, NamendaXR^®^, and Namzaric^®^). We showed that memantine preferentially blocks excessively/tonically activated (predominantly extrasynaptic) NMDA receptor-operated channels, while relatively sparing the phasic/physiological activity of synaptic NMDA receptor-associated channels (Chen et al., 1992; Chen and Lipton, 1997; Lipton, 2006, 2007; Xia et al., 2010; Emnett et al., 2013; Talantova et al., 2013; Takahashi et al., 2015). Memantine exerts this unique feature in part through its action as an open-channel blocker in which the drug preferentially blocks channels only when they are excessively/tonically open (Chen et al., 1992; Chen and Lipton, 1997; Lipton, 2006, 2007; Talantova et al., 2013; Molokanova et al., 2014). This mechanism represents a form of uncompetitive antagonism of the receptor, whereby a fixed concentration of memantine (e.g., 1 μM) blocks an increasing concentration of agonist (i.e., glutamate) to a greater extent than a lower concentration of agonist – a seemingly paradoxical finding but well known by pharmacologists for uncompetitive inhibitors (Lipton, 2006, 2007). Additionally, memantine has a relatively fast off-rate from the channels at physiological resting potential, preventing accumulation of the drug in the channel and thus maintaining normal synaptic function. Accordingly, unlike other potent NMDA receptor antagonists that persistently block channel activity and cause severe side effects, memantine relatively spares synaptic transmission required for normal cognitive function (Lipton, 2006, 2007). This mechanism of action (MOA) of memantine-like drugs, encompassing Uncompetitive, relatively Fast Off-rate, has thus been designated as the ‘UFO’ MOA. Nonetheless, one potential issue with memantine concerns its positive charge and therefore repulsion from channels after excessive influx of cations into sick neurons with consequent depolarization.

To overcome this issue, we recently developed modified aminoadamantane compounds (the class of drug that memantine falls into), including a lead drug candidate called NitroSynapsin (a.k.a. NitroMemantine, YQW-036, EM-036) (Lipton, 2006; Wang et al., 2006; Talantova et al., 2013; Takahashi et al., 2015; Ghatak et al., 2021a). These compounds afford improved efficacy via a dual inhibitory mechanism of NMDA receptors. They first block excessively-open channels using a memantine-like moiety, which then provides specific targeting of a nitro group ‘warhead’ to the S-nitrosylation sites on the NMDA receptor to provide additional inhibition (Figure 2). Importantly, this SNO-dependent antagonist function is not influenced by neuronal depolarization because the S-nitrosylation sites on the NMDA receptor are all located outside of the transmembrane domain and thus exterior to the voltage field sensed by the channel (Lei et al., 1992; Lipton et al., 1993; Choi et al., 2000; Lipton, 2006; Wang et al., 2006; Talantova et al., 2013; Takahashi et al., 2015). Thus, the dual inhibitory mechanism of NitroSynapsin offers dramatically improved efficacy in suppressing hyperexcitability and downstream aberrant NO production with consequent synaptic damage in both our animal models and in hiPSC-neuronal model systems, including cerebral organoid models of AD (Lipton, 2006; Wang et al., 2006; Talantova et al., 2013; Takahashi et al., 2015; Tu et al., 2017; Ghatak et al., 2021a). Moreover, our approach to specifically target an NO group (or more properly NOx, with x = 1 or 2) to NMDA receptor nitrosylation site(s) avoids *(i)* systemic side effects of NO, including systemic drop in blood pressure and, *(ii)* molecularly, off-target S-nitrosylation events on other proteins, such as Drp1 (Takahashi et al., 2015; Ghatak et al., 2021a). It should also be noted that NitroSynapsin is an aminoadamantane nitrate that donates a nitro group from an alkyl nitrate to S-nitrosylate NMDA receptors, instead of free radical NO*^•^*, thus lacking capability to produce neurotoxic free radical groups.

To show that NitroSynapsin decreases the hypersynchronous neural network activity and synaptic damage in a human context, we recently employed patch-clamp electrophysiology, calcium imaging, and multielectrode array (MEA) recordings in hiPSC-derived 2D neuronal cultures and 3D cerebral organoids (Ghatak et al., 2021a). We initially demonstrated that hiPSC-derived cerebrocortical neurons bearing familial AD mutations in APP or PS1 manifest high basal levels of intracellular calcium and increased spontaneous activity compared to their wild-type isogenic controls (Ghatak et al., 2019). As discussed above, this hyperexcitability is associated with non-convulsive epileptic events and cognitive impairment in patients with AD (Vossel et al., 2013; Lam et al., 2017). Critically, using our hiPSC neuronal model system, we found that NitroSynapsin significantly rebalanced aberrant neural network activity in AD neurons far more effectively than memantine, while sparing normal synaptic transmission in wild-type neurons (Ghatak et al., 2019). We had previously shown that NitroSynapsin attenuated aberrant electrical activity in the hAPP-J20 AD transgenic mouse model (Talantova et al., 2013), which is known to exhibit hyperexcitability if untreated (Palop et al., 2007). More recently, we also found in hAPP-J20 AD transgenic mice that treatment with NitroSynapsin produced S-nitrosylation of GluN1 subunits of the NMDA receptor and protected dendritic networks as well as presynaptic structures (Ghatak et al., 2021a). Hence these results are consistent with the premise that NitroSynapsin exerts a synapse protective function via selectively blocking excessively activated NMDA receptors and abrogating neuronal hyperexcitability. Interestingly, NitroSynapsin is also effective in rebalancing E/I abnormalities in autism spectrum disorder without significant side effects in multiple animal models, (Tu et al., 2017; Okamoto et al., 2019) and is moving toward human clinical trials slated for early next year.

Interestingly, aminoadamantane-like compounds, which would include memantine and NitroSynapsin, have been shown to improve E/I imbalance, and gamma band power and phase locking (Martina et al., 2013; Light et al., 2017). This raises the possibility that spectral frequency (1/f) analysis of EEGs can serve as a biomarker for target engagement and possibly as a surrogate for efficacy in future clinical trials.

Additionally, we have developed a series of other nitro-aminoadamantane compounds, one of which blocks the viroporin ion channel of SARS-CoV-2 and subsequently donates its nitro group to the endogenous viral receptor, ACE2, as the virus approaches the receptor; this blocks binding of the virus to the ACE2 and thus suppresses infection and spread of COVID-19 in the Golden Syrian hamster model in the absence of major side effects (Oh et al., 2022b). Taken together, these findings are consistent with the notion that specific targeting of SNO to particular proteins represents a viable path toward drug development. This type of drug design might be suitable for clinical regulation of specific SNO-proteins.

### 5.2. Other S-nitrosylation reactions that affect glutamate signaling and hyperexcitability in AD

As discussed above, multiple molecular mechanisms drive neuronal hyperexcitability in AD. Moreover, hyperexcitability is one of the primary drivers of aberrant protein S-nitrosylation in neurons (Lipton and Rosenberg, 1994; Talantova et al., 2013); neuroinflammation is another driver, affecting protein S-nitrosylation also in glial cells such as astrocytes and microglia (Yang et al., 2022) S-Nitrosylation can affect the activity of many hundreds or possibly thousands of proteins. Along these lines, in addition to modulating NMDA receptor activity, S-nitrosylation can regulate a whole series of proteins that are known to be associated with hyperexcitability. For example, protein S-nitrosylation can modulate glutamate release and clearance, as well as glutamate receptor expression, each of which contributes to glutamate signaling. Below, we summarize evidence for additional SNO-proteins involved in glutamatergic signaling and evaluate the potential influence of these SNO-proteins on neuronal hyperexcitability.

**FIGURE 3:**
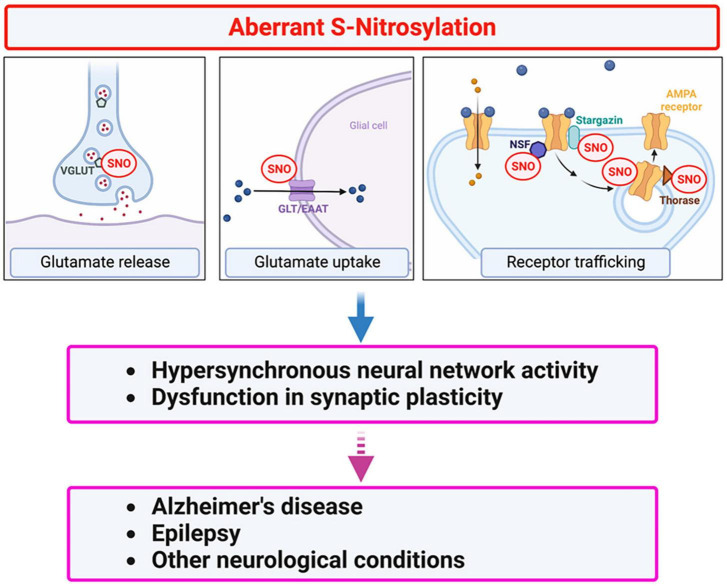
Protein S-nitrosylation regulates glutamatergic signaling contributing to hyperexcitability in AD. Excessive production of NO-related species due to hyperactivation of NMDA-type glutamate receptors results in S-nitrosylation (SNO) of multiple proteins. These include several proteins involved in glutamate release and re-uptake as well as AMPA receptor trafficking. For example, in addition to regulating NMDA receptor activity (see Figure 2), NO-mediated S-nitrosylation can regulate glutamatergic signaling via *(i)* vesicular glutamate transporter 1 (VGLUT1) involved in glutamate release, *(ii)* glutamate transporter-1 (GLT-1)/excitatory amino-acid transporter 2 (EAAT2) involved in glutamate clearance, and *(iii)* AMPA receptor subunits (e.g., GluA1) as well as AMPA receptor-associated proteins, such as stargazin, N-ethylmaleimide sensitive factor (NSF), and Thorase, involved in AMPA receptor recycling. Dysfunction in glutamate signaling can contribute to hyperexcitability and impaired synaptic plasticity in AD and other neurological conditions.

As alluded to above, in addition to mediating normal excitatory neurotransmission, glutamate signaling, mediated primarily through NMDA receptor overactivation, can lead to abnormal activity, contributing to hyperexcitability and eventual cognitive decline in disorders such as AD. To prevent this dysfunction, glutamate concentrations at the synaptic cleft must be tightly regulated both spatially and temporally via controlled glutamate release from the neuronal presynaptic terminal followed by rapid clearance, mainly by astrocytes. Accordingly, EAATs in astrocytes and VGLUTs at the presynaptic region regulate glutamate uptake and release, respectively. Additionally, expression of surface glutamate receptors at the postsynaptic sites may also influence glutamate signaling (Huang et al., 2005; Selvakumar et al., 2009, 2013; Gan et al., 2015; Charsouei et al., 2020; Umanah et al., 2020). Below, we discuss potential roles of protein S-nitrosylation in glutamate release and clearance as well as trafficking and internalization of glutamate receptors that influence abnormal hypersynchronous network activity (Figure 3).

#### 5.2.1. SNO-GLT-1/EAAT2-mediated regulation of glutamate clearance

Astrocytes surrounding the synaptic junction are predominantly responsible for the clearance of glutamate through Na^+^-dependent glutamate transporters, such as GLAST in rodent and EAAT1 in human, and GLT-1 in rodent, known as EAAT2 in human (Lehre and Danbolt, 1998; Rose et al., 2018). In AD brains, EAAT1 and EAAT2 expression is significantly decreased (Jacob et al., 2007), possibly contributing to an increase in glutamate levels at both the synaptic cleft and perisynaptic area to trigger excessive activation of extrasynaptic NMDA receptors. Moreover, genetic deletion of the GLT-1 gene results in lethal epileptic seizures in mice (Tanaka et al., 1997), consistent with the notion that elevated levels of extracellular glutamate due to GLT-1 dysfunction contributes to hyperexcitability.

Using chemoselective agents for enrichment or probes specific for SNO-proteins coupled with mass spectrometry (MS), our colleagues and collaborators Harry Ischiropoulos at the University of Pennsylvania and Steven Tannenbaum at MIT have identified between 1,500 and 2,000 proteins that can be S-nitrosylated in normal or diseased brains, representing the S-nitrosoproteome. For example, the Ischiropoulos group identified > 250 S-nitrosocysteine residues that are significantly decreased in nNOS (or eNOS) deficient mouse brains (Raju et al., 2015). Notably, using mouse tissues, that study found that proteins involved in glutamate metabolism, such as GLT-1, glutamate dehydrogenase (GDH), mitochondrial aspartate aminotransferase (mAspAT), and glutamine synthetase (GS), are all S-nitrosylated in an nNOS-dependent manner. Moreover, our group, in collaboration with Harry Ischiropoulos’ laboratory found that S-nitrosylation of key proteins implicated in glutamate homeostasis, including EAAT2, are highly S-nitrosylated in human postmortem brains with HAND, which is known to be associated with hyperactivation of NMDA receptors (Lipton et al., 1991; Doulias et al., 2021). In cell-based experiments, NO donors S-nitrosylated mouse GLT-1 at Cys373 and Cys561 (Cys374 and Cys563 in human EAAT2) to inhibit glutamate uptake (Raju et al., 2015). These findings are consistent with the notion that S-nitrosylation of GLT-1/EAAT2 increases extrasynaptic glutamate concentration, contributing to excessive extrasynaptic NMDA receptor activity and thus to hyperexcitability. In the future, *in vivo* characterization of S-nitrosylated GLT-1/EAAT2 in disease models could explore the therapeutic potential of inhibiting formation of SNO-GLT-1/EAAT2.

More recently, in collaboration with Steven Tanenbaum’s group, using a SNO-selective probe known as *SNO*TRAP coupled with MS analysis, we have detected nearly 1,500 proteins that are S-nitrosylated in human AD and control brains of both sexes (Yang et al., 2022). In this dataset, we also observe SNO-EAAT2 in human AD brains and in aged control brains, suggesting that the effect of S-nitrosylation of EAAT2 may be relevant to the human condition as well.

#### 5.2.2. Potential role of SNO-VGLUT1 in glutamate release

Packaging glutamate into synaptic vesicles by different vesicular transporters (VGLUT1-3) on their surface represents a critical step in glutamate release from the presynaptic terminus of excitatory neurons (El Mestikawy et al., 2011). To carry glutamate as an anion into the synaptic vesicles, VGLUTs utilize a proton electrochemical gradient generated by vacuolar (H^+^) ATPases (Naito and Ueda, 1985). Once stored in synaptic vesicles, glutamate is released from presynaptic termini upon stimulation. Among VGLUT family members, in the adult brain, VGLUT1 is believed to be the main isotype, accounting for the majority of glutamate transport into synaptic vesicles at excitatory glutamatergic terminals (Du et al., 2020). Using hiPSC-derived cerebrocortical neuronal cultures, we recently found that both 2D cultures and 3D organoids bearing AD-linked mutations exhibit increased expression of VGLUT1 compared to isogenic wild-type controls; this increased expression of VGLUT1 may lead to increased release probability (Ghatak et al., 2019). In concordance with our hiPSC-based results, increased levels of VGLUT1 have also been found in human AD brain tissue and AD transgenic mouse models (Timmer et al., 2014; Wang et al., 2017). Coupled with our electrophysiological findings, these results suggest that aberrantly increased expression VGLUT1 may increase release probability to contributes to excitatory synaptic hyperactivity in AD.

Additionally, VGLUT1 has been shown to be S-nitrosylated under disease conditions (Wang et al., 2015, 2017). In fact, S-nitrosylation of VGLUT1 coincides with NO-mediated inhibition of vesicular uptake of glutamate, consistent with the premise that S-nitrosylation decreases VGLUT1 transport activity (Wang et al., 2015). In the APP/PS1 transgenic AD mouse model, S-nitrosylation of VGLUT1 is increased in the hippocampus at pre-symptomatic stages compared to control (Wang et al., 2017). However, when animals become cognitively symptomatic, total VGLUT1 expression increases while SNO-VGLUT1 levels decrease. These findings suggest that S-nitrosylation of VGLUT1 at the pre-symptomatic stage may limit excessive glutamatergic neurotransmission to delay the progression of pathological processes. To critically test this hypothesis, additional work will be needed. For example, future studies should determine the site(s) of S-nitrosylation on VGLUT1, show direct evidence that S-nitrosylation indeed inhibits glutamate uptake into vesicles, and mechanistically demonstrate that S-nitrosylation of VGLUT1 offers synaptic protection by decreasing release probability.

#### 5.2.3. Multiple SNO-proteins affect AMPA receptor activity and expression in the postsynaptic membrane

AMPA receptors can be calcium permeable or impermeable depending on subunit composition, and they display faster kinetics during neurotransmission than NMDA-type glutamate receptor-operated currents (Traynelis et al., 2010). AMPA receptors typically exist as homo- or hetero-tetramers of GluA1-4 subunits (Herguedas et al., 2016). Each subunit consists of an extracellular N-terminal domain, a ligand-binding domain, a transmembrane domain, and a cytoplasmic C-terminal domain. Additionally, AMPA receptor subunits are highly dynamic membrane proteins, continuously trafficking in and out of the postsynaptic membrane (Gan et al., 2015). Accordingly, the precise regulation of AMPA receptor trafficking and activity is crucial for excitatory neurotransmission and synaptic plasticity. In contrast, dysfunction in synaptic AMPA receptor activity or trafficking machinery can contribute to aberrant glutamate signaling, leading to neuronal hyperexcitability, cognitive impairment, and epileptic seizures (Charsouei et al., 2020). Of particular note, NO-related species can modulate surface expression and conductance of AMPA receptors via S-nitrosylation not only of the AMPA receptor itself but also AMPA receptor-associated proteins, including stargazin, N-ethylmaleimide sensitive factor (NSF), and Thorase (Matsushita et al., 2003; Huang et al., 2005; Selvakumar et al., 2009; Ho et al., 2011; Umanah et al., 2020).

Often AMPA receptors and NMDA receptors are expressed at the same postsynaptic sites. Activation of NMDA receptors can augment AMPA receptor conductance, at least in part, via increased CaMKII-mediated phosphorylation of Ser831 on the GluA1 subunit (Barria et al., 1997). On the other hand, stimulation of NMDA receptors can also lead to increased endocytosis of AMPA receptors, resulting in removal of AMPA receptors from postsynaptic membranes, consequently associated with decreased synaptic strength and contributing to the development of LTD (Man et al., 2000). Several studies have demonstrated that NO-related species produced by NMDA receptor activation can increase S-nitrosylation of AMPA receptor GluA1 subunits at Cys875 (Cys893 if including the signal peptide on the N-terminus). This S-nitrosylation reaction enhances Ser831 phosphorylation on GluA1 (Selvakumar et al., 2013; von Ossowski et al., 2017), but the precise mechanism for this effect remains unknown. Furthermore, non-nitrosylatable mutant GluA1 (i.e., substitution of Cys875 to Ser) was shown to abrogate the phosphorylation-dependent increase in single-channel conductance of GluA1 (Selvakumar et al., 2013). Intriguingly, non-nitrosylatable mutant GluA1 also decreases binding to AP2 protein, an adaptor complex important in clathrin-mediated endocytosis, thus diminishing internalization of AMPA receptors (Selvakumar et al., 2013). This result suggests that S-nitrosylation increases endocytosis of GluA1. Moreover, a recent study reported that GluA1 (Cys875) is also important for interaction with SAP97 (von Ossowski et al., 2017), a member of the membrane-associated guanylate kinase homologue (MAGUK) scaffolding proteins that play important roles in trafficking and membrane targeting of ion channels (Fourie et al., 2014). Whether S-nitrosylated GluA1 affects the direct interaction between GluA1 and SAP97 remains to be examined, however. In summary, these findings are consistent with the notion that S-nitrosylation of GluA1 at Cys875 facilitates activity-dependent phosphorylation, channel conductance, and eventually endocytosis of AMPA receptors, each of which could be important in controlling the hyperactivity that occurs in neurodegenerative disorders.

Regulation of AMPA receptor trafficking and membrane expression also depends on auxiliary subunits and the receptor-associated proteins (Henley et al., 2011; Greger et al., 2017). Notably, NO-related species can control cell surface AMPA receptor expression through S-nitrosylation of AMPA receptor-associated proteins, including stargazin, NSF, and Thorase (Huang et al., 2005; Selvakumar et al., 2009; Umanah et al., 2020). As an example, stargazin is an auxiliary subunit of AMPA receptors that upregulates surface expression of synaptic AMPA receptors. Endogenous NO from the activation of NMDA receptors can trigger S-nitrosylation of stargazin at Cys302, located at the C-terminus of the protein; this augments stargazin binding to GluA1, enhancing surface expression of this AMPA receptor subunit (Selvakumar et al., 2009). Moreover, since stargazin directly binds to all four AMPA receptor subunits through both intra- and extracellular domains, it is anticipated that SNO-stargazin can stabilize not only GluA1-containing receptors but a large fraction of AMPA receptors.

Secondly, NSF is an ATPase that functions as a SNARE chaperone to regulate vesicle transport in multiple cell types. In neurons, when NSF binds to GluA2, it interferes with the interaction of the SNARE complex to Pick1 (protein interacting with C-Kinase 1); this is known to promote AMPA receptor expression at the membrane surface and is therefore involved in maintaining the number of AMPA receptors at the synapses (Hanley, 2018). As its name suggests, NSF contains highly reactive sulfhydryl groups that are sensitive to N-ethylmaleimide (Block et al., 1988). Consistent with this notion, NSF contains reactive thiol groups, particularly at Cys residues 21, 91, and 264, that can be efficiently S-nitrosylated (Matsushita et al., 2003). Interestingly, in endothelial cells S-nitrosylation of NSF does not compromise its ATPase activity but does inhibit its disassembly with the SNARE complex, thus regulating vesicle transport (Matsushita et al., 2003). At neuronal synapses, S-nitrosylation of NSF augments its binding to GluA2, thus enhancing surface expression of GluA2 by disrupting GluA2-Pick interaction (Huang et al., 2005). Moreover, S-nitrosylation of NSF has been observed under conditions that induce synaptic plasticity, suggesting that SNO-NSF may contribute to LTP (Huang et al., 2005).

The last example described here is S-nitrosylation of Thorase (Umanah et al., 2020). Thorase is an AAA^+^ (ATPases Associated with diverse cellular Activities) ATPase that facilitates endocytosis and internalization of AMPA receptors. Mechanistically, glutamate receptor–interacting protein 1 (GRIP1) binds to the C-terminus of GluA2 and drives surface expression of AMPA receptor. In contrast, Thorase mediates the disassembly of the AMPA receptor-GRIP1 complex in an ATP-dependent manner, thus increasing internalization of AMPA receptors (Zhang et al., 2011). Accordingly, knockdown or knockout of Thorase results in increased surface postsynaptic AMPA receptor expression, potentially contributing to hyperexcitability and epilepsy (Zhang et al., 2011). A recent study demonstrated that NMDA receptor activation leads to S-nitrosylation of Thorase at Cys137 (Umanah et al., 2020). S-Nitrosylation of Thorase stabilizes AMPA receptor-Thorase complexes, thereby inhibiting surface expression of the receptor. As a possible negative-feedback mechanism, however, S-nitrosylated Thorase can also transnitrosylate NSF, triggering SNO-NSF-mediated upregulation of AMPA receptors at the postsynaptic membrane, as described above. Importantly, non-nitrosylatable mutant Thorase significantly decreases SNO-NSF formation and synaptic expression of GluA2 (Umanah et al., 2020), indicating the possible pathophysiological relevance of SNO-Thorase in AMPA receptor expression and thus electrical activity. Finally, expression of non-nitrosylatable mutant Thorase also causes impairment in both LTP and LTD. Thus, these findings are consistent with the notion that S-nitrosylated Thorase, in addition to SNO-NSF, modulates AMPA receptor trafficking and synaptic plasticity (Umanah et al., 2020).

Collectively, S-nitrosylation of AMPA receptor subunits and proteins that interact with the receptor can facilitate trafficking of AMPA receptors both in and out of the synapse, depending on the target of S-nitrosylation. For example, SNO-GluA1 and SNO-Thorase mediate internalization of AMPA receptors, whereas SNO-stargazin and SNO-NSF contribute to enhanced expression of AMPA receptors at the synaptic membrane surface. Notably, dysregulation of AMPA receptor trafficking and activity may contribute to neuronal hyperexcitability and cognitive impairment in neurodegenerative disorders such as AD. Thus, future investigations should include spaciotemporal comparisons of these known SNO-proteins during various stages of AD in experimental model systems and in human AD postmortem brains.

## 6. Concluding remarks

Since neuronal hyperexcitability is an early phenotype observed both in human AD patients and in experimental models of AD, including hiPSC-derived 2D cultures and 3D cerebral organoids, it is important to understand the pathological effect of hyperexcitability on AD brain. Glutamate accumulation and dysregulated glutamate receptor function have been shown to contribute to hyperexcitability-related pathology, including synaptic loss. Glutamate receptors/transporters involved in release, uptake, and postsynaptic signaling contribute to this excitotoxic synaptic damage. Several studies and reviews have delineated aberrant hyperactivity both at the single neuron level and at the neural network level. It is well known that one of the major features of neurodegenerative diseases like AD is excessive accumulation of ROS/RNS, in part due to hyperactivity of NMDA receptors. Excessive ROS and RNS affect the function of many cellular molecules and can interfere with cellular signaling. In particular, reactions of high levels of NO-related species, resulting in aberrant protein S-nitrosylation, can feedback to lead to further hyperactivity and synaptic damage, affecting normal brain function. However, there is a lack of comprehensive literature on how ROS or RNS-dependent posttranslational modifications are affected by excessive glutamate signaling and, in turn, contribute to neuronal hyperexcitability. Here, we review the involvement of redox-mediated posttranslational modifications such as protein S-nitrosylation, in part triggered by excessive glutamate receptor activity, causing additional changes in glutamate receptors and other deleterious pathways. Other pathways disrupted by aberrant protein S-nitrosylation, but beyond the scope of the current review, include autophagy, which would otherwise clear misfolded/aggregated proteins, other protein folding machinery, chaperone activity, metabolism needed for synaptic maintenance, and many other cellular processes (Uehara et al., 2006; Nakamura et al., 2013, 2021c; Nakamura and Lipton, 2017; Kim et al., 2022; Oh et al., 2022a). Taken together, these events result in hyperexcitability contributing to synaptic damage. We also discuss how candidate therapeutic agents like NitroSynapsin, acting via a physiological negative-feedback mechanism mediated by selective S-nitrosylation of NMDA receptors, can decrease hyperexcitability and ameliorate synaptic loss. These findings highlight the potential application of specifically-targeted protein S-nitrosylation (or potentially denitrosylation of other proteins) as a therapeutic strategy in AD. As future directions for research, it will be important to identify additional aberrant S-nitrosylated or transnitrosylated protein networks that contribute to hyperexcitability and synaptic damage. One type of experimental approach for this purpose is mass spectrometry-based S-nitrosoproteomic analyses together with bioinformatic techniques to uncover comprehensive information on S-nitrosylated and transnitrosylated proteins involved in aberrant glutamate and other downstream signaling pathways that mediate synaptic damage (Nakamura et al., 2021a,b; Yang et al., 2022). Hence, additional studies to elucidate the causal role(s) of protein S-nitrosylation in neuronal network impairment may identify potential targets for therapeutic intervention in order to prevent the hypersynchronous neural network activity in AD and other neurological conditions.

## Author contributions

SG and TN: conceptualization, original draft—writing and editing, and figure construction. TN and SL: conceptualization, manuscript editing, funding acquisition, and final approval of the manuscript. All authors contributed to the article and approved the submitted version.
